# Comparison of a novel hand-held retractor-assisted transforaminal lumbar interbody fusion by the wiltse approach and posterior TLIF: a one-year prospective controlled study

**DOI:** 10.1186/s12891-024-07248-w

**Published:** 2024-02-14

**Authors:** Xing Shen, Fu Tao Li, Yong Quan Cheng, Ming Hui Zheng, Xin Qiang Yao, Hai Ming Wang, Jian Ting Chen, Hui Jiang

**Affiliations:** 1 Division of Spinal Surgery, Department of Orthopaedics, Nanfang Hospital, Southern Medical University, 1838 North Guangzhou Avenue, Guangzhou, China; 2 Center for Orthopaedic Surgery, The Third Affiliated Hospital of Southern Medical University, Guangzhou, China

**Keywords:** Wiltse approach TLIF, Open, Transforaminal lumbar interbody fusion, Hand-held retractors system

## Abstract

**Background:**

This study aims to compare the clinical outcomes and safety of a novel hand-held retractor system-assisted Wiltse TLIF with that P-TLIF and assess whether this hand-held retractor system assisted Wiltse TLIF can yield less paraspinal muscle injury.

**Methods:**

56 patients (P-TLIF: 26, Wiltse TLIF: 30) were included in this one year prospective controlled study. The operation time, intraoperative blood loss, postoperative drainage, mobilization time, and discharge time were recorded. The clinical outcomes were evaluated by ODI, VAS, JOA, and SF-36 scores (7 days, 3, 6, and 12 months after surgery). Paraspinal muscle injury was assessed by postoperative MRI (6 months after surgery). CK and C-reaction protein were measured pre and postoperatively, and CT or X-ray (one year postoperatively) was used to assess bony union/non-union.

**Results:**

The Wiltse (study) group was associated with significantly less estimated blood loss (79.67 ± 28.59 ml vs 192.31 ± 59.48 ml, *P* = 0.000*), postoperative drainage (43.33 ± 27.89 ml vs 285.57 ± 123.05 ml, *P* = 0.000*), and shorter mobilization (4.1 ± 1.2 d vs. 3.0 ± 0.9 d, *P* < 0.05) and discharge times (7.7 ± 1.9 d vs. 6.1 ± 1.2 d, *P* = 0.002*) than the P-TLIF (control) group. Serum CK activity at 24 h postoperatively in the study group was significantly lower than in the control group (384.10 ± 141.99 U/L vs 532.76 ± 225.76 U/L, *P* = 0.018*). At 7 days after surgery, VAS (2.3 ± 0.6 vs 3.2 ± 0.7, *P* = 0.000*)and ODI scores (43.9 ± 11.9 vs 55.2 ± 12.9, *P* = 0.001*) were lower, while the JOA scores (18.4 ± 3.4 vs 16.3 ± 4.2, *P* = 0.041*) was higher in the control group than in the study group. Results observed at 3 months of follow-up were consistent with those at 7 days. After six months postoperatively, paraspinal muscle degeneration in the control group was more significant than in the study group (*P* = 0.008*).

**Conclusion:**

Our study showed that this novel hand-held retractor system assisted Wiltse approach TLIF can significantly reduce paraspinal muscle injury, postoperative drainage, and intraoperative blood loss, mobilization and discharge time, as well as yield better short-term outcomes compared to P-TLIF.

**Trial registration:**

25/09/2023 NCT06052579.

## Background

Posterior transforaminal lumbar interbody fusion (P-TLIF) has been performed for many years to fuse and stabilize the spine, yielding excellent results [1]. However, P-TLIF requires significant tissue dissection, and many researchers have put forward that excessive paraspinal muscle dissection and retraction can have deleterious effects during lumbar procedures [2, 3]. During surgical procedures, the over dissection of the paraspinal muscles can result in more intraoperative bleeding, postoperative drainage, and even postoperative paraspinal muscle degeneration. Paraspinal muscles play an important role in maintaining lumbar segmental stability, and recent studies have shown that atrophy of paraspinal muscles is a significant cause of intractable lumbar pain following surgery which seriously affects the quality of life of the patients [4]. Therefore, reducing paraspinal muscles injury caused by lumbar fusion surgery has become a focus of attention among many surgeons.

With the development of minimally invasive spine surgery, Foley and Smith first introduced minimally invasive TLIF (MIS-TLIF), using a tubular retractor system to perform spinal canal decompression through the gap between the multifidus and the longissimus (Wiltse approach) [5]. Compared with P-TLIF, MIS-TLIF can reduce paraspinal muscle damage, intraopreative blood lose, postoperative drainage and lead to better postoperative clinical outcomes, which has been extensively documented in the literature [6]. However, some scholars have cautioned that the use of the tubular retractor system for spinal canal decompression via the Wiltse approach may potentially capture a portion of the paraspinal muscles inside the retractor, causing disruption of the surgical field [7]. As a result, the surgeon may have to remove the paraspinal muscles from the retractor, which can increase the likelihood of iatrogenic injury to the paravertebral muscles.

In recent years, hand-held retractors have been applied to assist in Wiltse approach to perform canal decompresion, causing less paraspinal muscle injury and yielding better postoperative clinical outcomes than P-TLIF [8, 9]. However, few prospective studies have been conducted comparing the clinical and radiological outcomes between Wiltse TLIF and P-TLIF, both assisted by hand-held retractors. Therefore, further research is warranted to assess whether hand-held retractor-assisted Wiltse TLIF can yield less paraspinal muscle injury and better postoperative clinical outcomes.

**Fig. 1:**
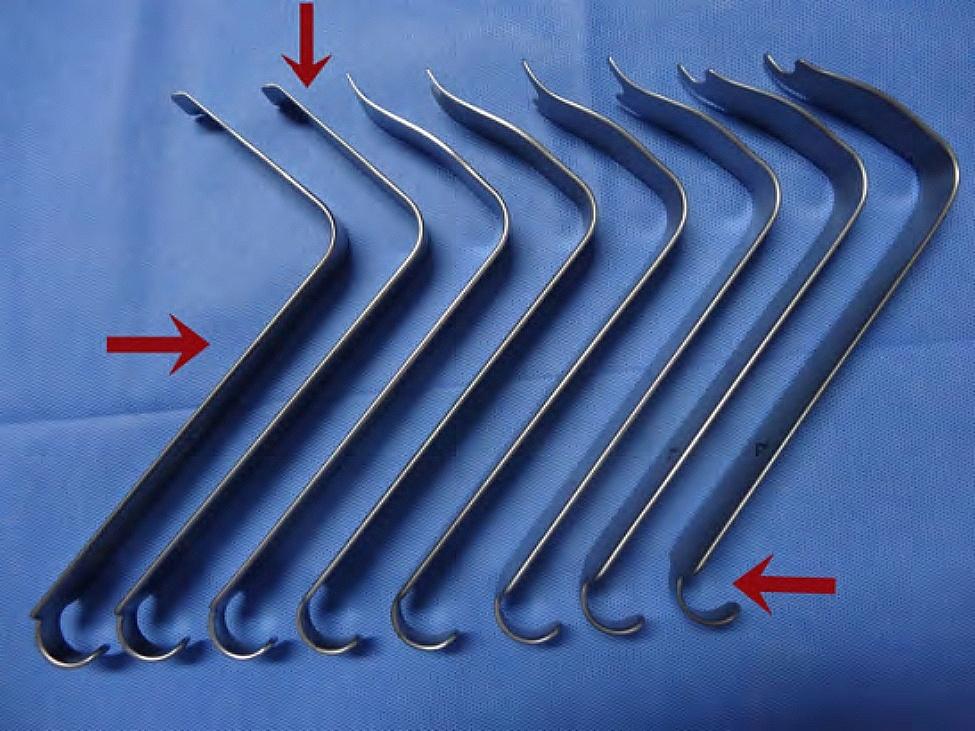
Novel hand-held retractor system (↓: top part; →body part; ←end part)

In present study, we introduce a new type of hand-held retractor system (Fig. 1) primarily utilized for the Wiltse approach TLIF. The aims of this study was to compare the clinical outcomes and safety of this retractor-assisted WiltseTLIF with that of P-TLIF and assess whether this novel hand-held retractor-assisted Wiltse TLIF can yield less paraspinal muscle injury.

## Methods

This one-year prospective controlled study, comparing two approaches (Wiltse TLIF vs. P-TLIF) for hand-held retractors-assisted TLIF, was approved by the Institutional Review Board (IRB) of our hospital and has registered on ***clinical trial. Gov(25/09/2023 NCT06052579)***. Written informed consent was obtained from all study participants.From August 2016 to February 2017, 56 eligible patients were included in this study and were divided into two groups. 30 patients (18 males, 12 females) were included in the study group and underwent Wiltse TLIF, and 26 (16 males, 10 females) were included in the control group and underwent P-TLIF. All patients underwent one or two-level spinal fusion surgery due to lumbar stenosis, grade 1 or 2 spondylolisthesis, or lumbar disc herniation with lumbar instability and mechanical lower back pain. The types of surgery were not completely randomly assigned and were determined by the time of visit. Patients with lumbar infection (i), spinal tumor (ii), severe osteoporosis (iii), pregnant and lactating women (iv), severe lumbar stenosis (v), and severe comorbidities (vi) were excluded from this study.

### Description of new hand-held retractor system

**Fig. 2:**
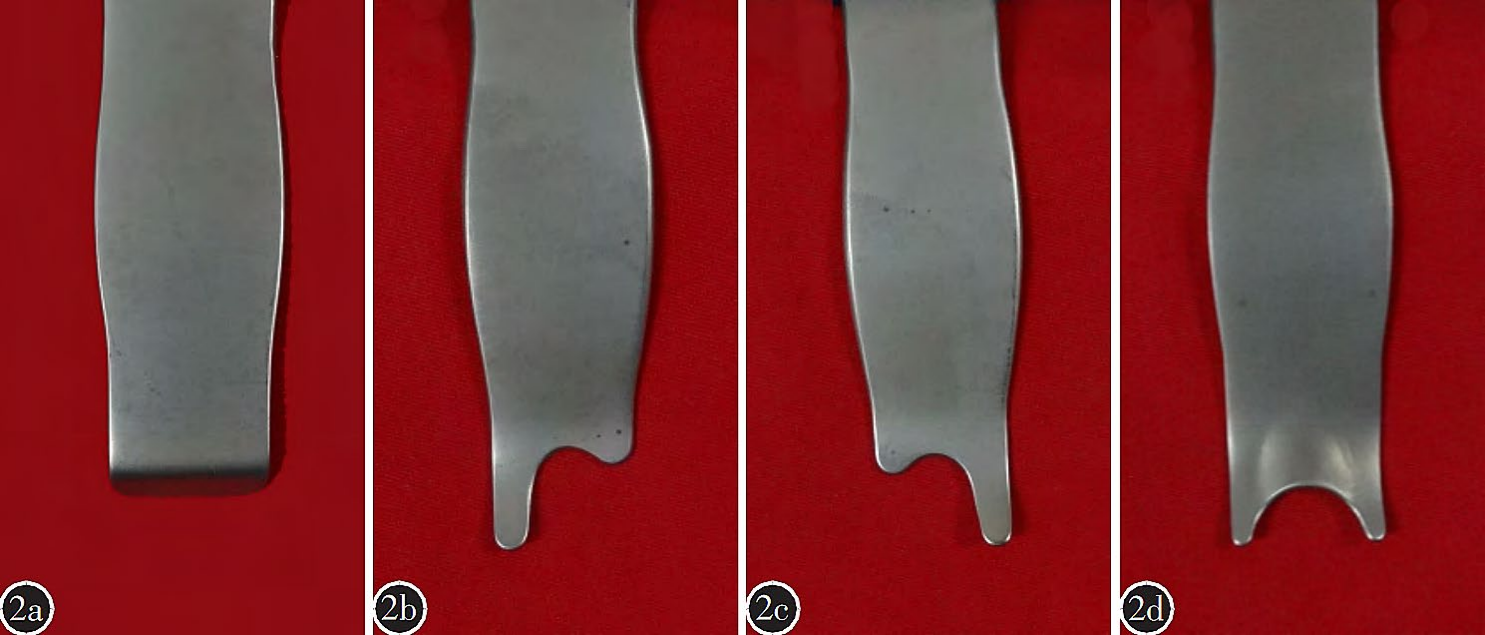
**a**:top part of tissues retractor; **b** and **c**:top part of articular process retractor; **d**:top part of pedicle screw

According to their functions, the novel hand-held retractor system can be divided into soft tissue retractor, articular process retractor, and pedicle screw retractor (Fig. 2).

**Fig. 3:**
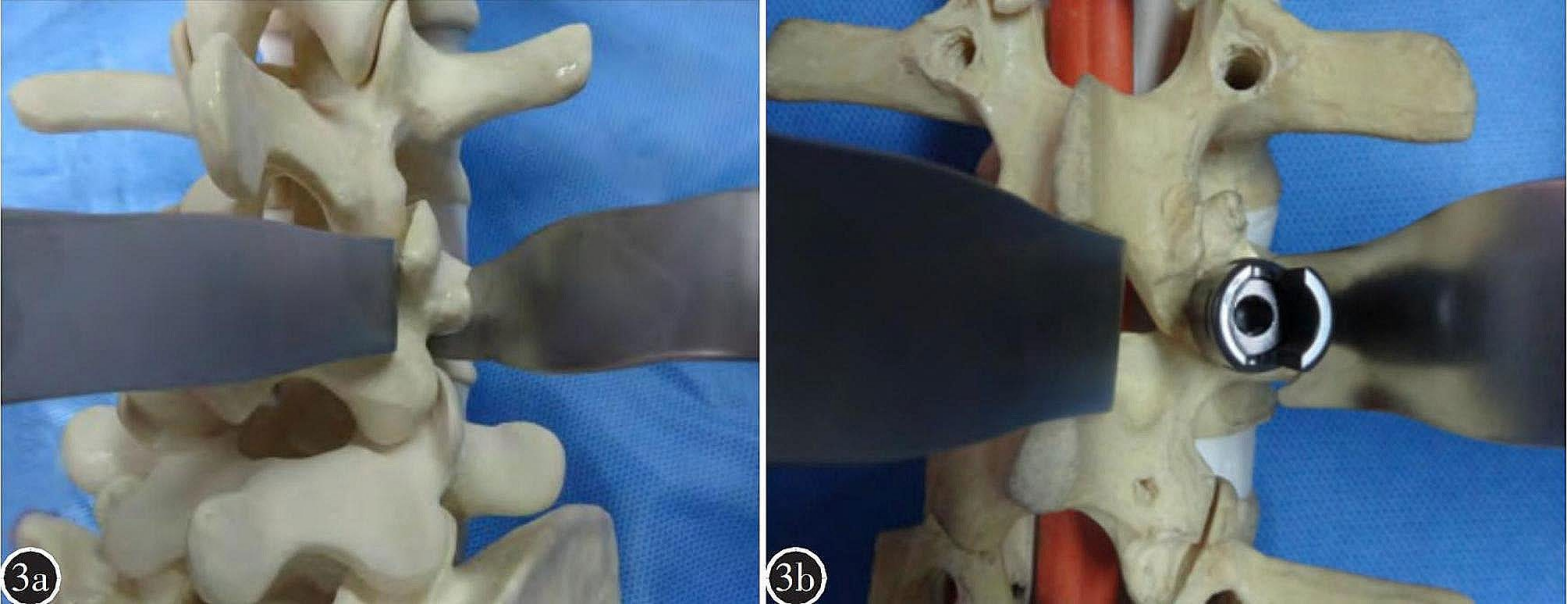
Illustration of the usage for this novel hand-held retractor system: **a**: the left is tissue retractor and the right is articular process retractor **b**:right is the pedicle screw retractor

1. Soft tissue retractor: The head of the soft tissue retractor (Fig. 2a) is flat, exhibiting a 30° inward angle within the initial 3 mm. The body of the soft tissue retractor is flat and takes on a “7” shape, while the tail forms a pointed hook, curving upward in a semi-circular shape. The soft tissue retractor is mainly used to pull the multifidus muscle inward and longissimus muscle outward.

1. Articular process retractor: The head of the articular process retractor has long and short arms in an “L” shape, with a 45° outward angle within the initial 5 mm. The body of the articular process retractor is flat and takes on a “7” shape, while the tail forms a pointed hook, curving upward in a semi-circular shape. The “L”- shaped top part of articular process retractor comprises a short arm and a long arm, the pedicle screw entry point of lumbar veterbrae can be exposed by clamping its short arm at the basement of the transverse process and the long arm at the lower edge of the transverse process. The pedicle screw entry point of S1 can also be exposed by clamping its short arm at the lateral margin of the superior articular process and the long arm at the sacrum (Fig. 3a).
2. Pedicle screw retractor: The top part of pedicle screw retractor is “U” - shaped with a 45° outward angle within the first 5 mm. The body of pedicle screw retractor is flat and takes on a “7” shape, while the tail forms a pointed hook, curving upward in a semi-circular manner., the surgical field can be exposed by clamping the “U” shaped head at the base of pedicle screw during theprocedure.(Fig. 3b).

### Assessment of preoperative data

Preoperative data including patients’ age, gender, anterior-posterior, lateral and dynamic (flexion/extension) plain lumbar spine radiography, MRI, and/or CT. Visual analog scale (VAS), Oswestry disability index (ODI) SF-36 quality of life and JOAscores were obtained from all patients(). The SF-36 measures eight scales: physical functioning (PF), role physical (RP), bodily pain (BP), general health (GH), vitality (VT), social functioning (SF), role emotional (RE), and mental health (MH). In addition, the serum creatine kinase (CK) and C-reactive protein (CRP) were measured preoperatively.

## Surgical procedure

### Study group

**Fig. 4:**
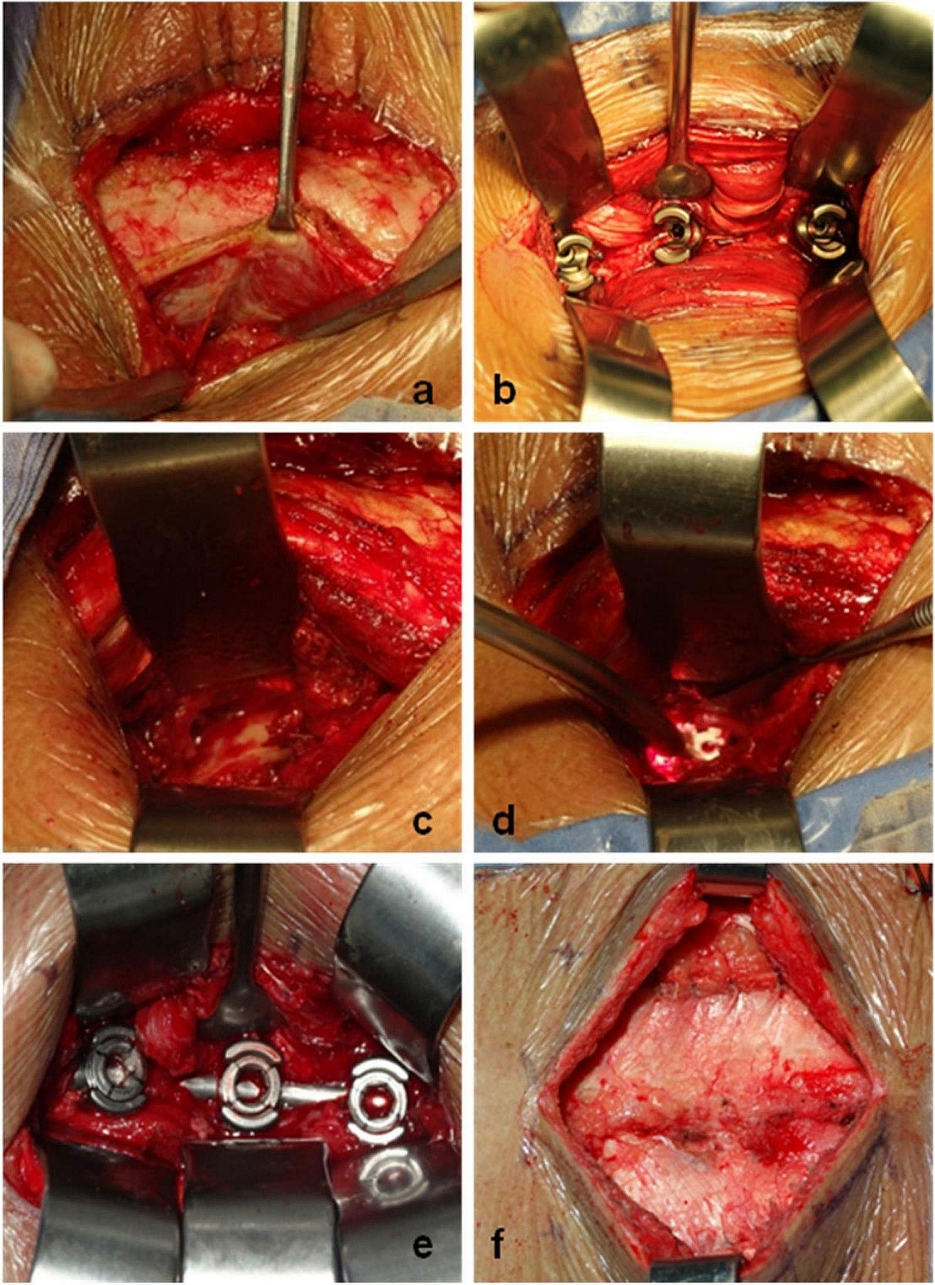
Hand-held retractors system assisted Wiltse approach TLIF, **a**: exposure; **b**: screw-setting; **c**: decompression; **d**: fusion; **e**: rod-setting; **f**: suture

A posterior midline skin incision was made, and the skin and subcutaneous tissue were incised to expose the lumbodorsal fascia. On the normal side, the lumbodorsal fascia was split to form a gap between the multifidus and longissimus muscles (Fig. 4a). The inferior facet joint was exposed, and pedicle screws were placed with the help of the articular process retractor (Fig. 4b) under direct vision. On the symptomatic side, the above steps were repeated to place the cephalad pedicle screws, prepare the caudal pedicle screw channel and expose the inferior facet joint and part of the vertebral veterbral plate inside the screw channel. The multifidus muscle was pulled inward using the tissue retractor while clamping the screw pedicle retractor at the base of pedicle screw, then the veterbral plate is exposed. Next the lateral edge of the veterbral plate was dissected to expand the intervertebral foramen (Fig. 4c), expose the nerve root and dural sac, remove the herniated nucleus pulposus and excise the cartilaginous endplate. A cage filled with local bone was inserted into the disc space restoring the spacing between the vertebrae (Fig. 4d), and was finally locked into place using a screw and rod system (Fig. 4e). Finally, a drainage tube was placed through the incision on the decompression side,and Stitched the incision layer-by-layer (Fig. 4f).

### Control group

A posterior midline incision was made directly over the lumbar vertebra to be fused, and paraspinal muscles were dissected from the spinous process, veterbral plate. Then the vertebral veterbral plate and lateral aspect of the facet joint were exposed, and bilateral pedicle screws were inserted. On the symptomatic side, the lower half part of the superior veterbral plate and the upper half of the inferior veterbral plate were dissected, followed by an incision of the ligamentum flavum to expose the nerve root, the dural sac and the intervertebral disc. Then the nucleus pulposus was removed, and the cartilaginous endplate was excised. A cage filled with local bone was inserted into the disc space restoring the spacing between the vertebrae, and was finally locked into place using a screw and rod system. Finally, a drainage tube was placed through the incision on the decompression side,and Stitched the incision layer-by-layer.

### Assessment of perioperative and follow-up time parameters

The perioperative and follow-up time data of all patients were collected, including the operation time, intraoperative blood loss, postoperative drainage, mobilization time, and discharge time. The CK and CRP levels were measured 24 h after surgery. All patients were evaluated by independent surgeons using VAS, ODI, and JOA scores during the follow-up (7 days, 3, 6, and 12 months after surgery).

In this study, the method described by Kader D. F [10]. was used to assess the fatty infiltration and fibrosis severity of affected layers based on lumbar MRI at 6 months after surgery. At one year postoperatively, lumbar CT or anteroposterior-lateral lumbar X-ray was used to assess bone healing according to the method described by Bridwell [11].

### Statistical analysis

A student’s t-test was used to compare measurement data that conformed to normal distribution and homogeneity of variance. A paired t-test was used to compare the measurement data of the same patient before and after surgery. Classification data were compared by χ [2] tests. When the sample size was less than 40 or the expected or theoretical frequency in any cells was less than 1, Fisher’s exact test was adopted. The Mann-Whitney U test was used to assess data that did not conform to normal distribution. When there were significant differences between groups, the LSD method was used for pairwise comparison. A *P*-value < 0.05 was statistically significant.

## Results

### Demographic

The mean age of the control and study groups was 53.2 ± 11.3 and 54.0 ± 12.1, respectively. There was no significant difference between the two groups in age, gender and operated segments (*P* > 0.05).

### Perioperative metric

The operation time was comparable between the control and study groups (171.73 ± 34.25 min vs. 179.60 ± 21.77 min, *P* = 0.289).The intraoperative blood loss and postoperatibe showed significant differences between two groups.The study group was associated with significantly less intraoperative blood loss (79.67 ± 28.59 ml vs. 192.31 ± 59.48 ml, *P* = 0.000*) and postoperative drainage (43.33 ± 27.89 ml vs. 285.57 ± 123.05 ml, *P* = 0.000*) than the control group. In addition, the study group was associated with significantly shorter mean mobilization time (4.1 ± 1.2 d vs. 3.0 ± 0.9 d, *P* = 0.000*) and discharge time (7.7 ± 1.9 d vs. 6.1 ± 1.2 d, *P* = 0.002*) compared to the control group (Table 1).

**Table 1:** The demographic and clinical characteristics and perioperative data of the patients of P-TLIF and wiltse approach TLIF

	P-TLIF	Wiltse approach TLIF	*P* Value
No. of patients	26	30	
Sex (M/F)	16/10	18/12	*P* = 0.906
Age, yr	53.2 ± 11.3	54.0 ± 12.1	*P* = 0.808
Operation segment			
L4-L5	11	19	-
L5-S1	7	3	-
L3-L5	4	3	-
L4-S1	4	5	-
Operation time, min/segment	171.73 ± 34.25	179.60 ± 21.77	*P* = 0.289
Estimate blood loss, mL/segment	192.31 ± 59.48	79.67 ± 28.59	*P* = 0.000*
postoperative ambulation time,d	4.1 ± 1.2	3.0 ± 0.9	*P* = 0.000*
Discharge time, d	7.7 ± 1.9	6.1 ± 1.2	*P* = 0.002*
Postopreative drainage, ml	285.57 ± 123.05	43.33 ± 27.89	*P* = 0.000*
Serum creatine kinase, IU/L			
Preoperatively	84.53 ± 37.52	74.75 ± 24.73	*P* = 0.349
1 day postoperatively	532.76 ± 225.76	384.10 ± 141.99	*P* = 0.018*
C relative protein(mg/L)			
Preoperatively	1.72 ± 0.33	1.71 ± 1.14	*P* = 0.980
1 day postoperatively	28.95 ± 24.32	29.50 ± 13.27	*P* = 0.754

### Evaluation of paravertebral muscle injury

There was no significant difference between the two groups in peripheral blood serum CRP concentrations and CK activity before surgery (*P* > 0.05). A significant increase in serum CRP concentration and CK activity was observed 24 h after the operation. However, the postoperative 24 h serum CK activity in the study group was significantly lower than the control group (384.10 ± 141.99 U/L vs. 532.76 ± 225.76 U/L, *P* = 0.018*), and the postoperative 24 h serum CRP concentration remained comparable (Table 1).

### VAS scores

There were no significant differences in VAS scores between two groups before the operations (5.96 ± 1.6 VS 5.73 ± 1.6; *P*>0.05), at 7 days after the operation, the study group was associated with significantly lower VAS (2.3 ± 0.6 vs 3.2 ± 0.7; *P* = 0.000*); The results at 3 months postoperatively were consistent with the results at 7 days postoperatively (1.43 ± 0.97 VS 2.38 ± 0.63; *P* = 0.000*), however, there was no significant difference between the two groups in VAS scores at 6 and 12 months (Table 2).

**Table 2:** Preopreative and follow-up time VAS、ODI、JOA scores

	peropreative	7 days	3 months	6 months	1 year
VAS (P-TLIF)	5.96 ± 1.6	3.2 ± 0.7	2.38 ± 0.63	1.31 ± 1.07	0.88 ± 0.65
VAS (Wiltse approach TLIF)	5.73 ± 1.6	2.3 ± 0.6	1.43 ± 0.97	1.10 ± 0.88	0.70 ± 0.75
*P* value	0.605	0.000*	0.000*	0.434	0.334
ODI (P-TLIF)	56.8 ± 21.7	55.2 ± 12.9	29.46 ± 9.42	15.92 ± 7.65	14.19 ± 5.87
ODI (Wiltse approach TLIF)	54.1 ± 20.0	43.9 ± 11.9	16.13 ± 7.10	12.93 ± 4.39	13.30 ± 5.72
*P* value	0.630	0.001*	0.000*	0.074	0.568
JOA (P-TLIF)	11.8 ± 5.8	16.3 ± 4.2	21.77 ± 2.66	24.80 ± 3.14	25.65 ± 2.33
JOA (Wiltse approach TLIF)	12.8 ± 5.8	18.4 ± 3.4	23.83 ± 1.86	25.53 ± 1.11	26.10 ± 1.75
*P* value	0.51	0.041*	0.001*	0.241	0.418

### ODI scores

There were no significant differences in ODI scores between two groups before the operations (56.8 ± 21.7 VS 54.1 ± 20.0; *P*>0.05), At 7 days after the operation, the study group was associated with significantly lower ODI (43.9 ± 11.9 vs 55.2 ± 12.9; *P* = 0.001*). The results at 3 months postoperatively were consistent with the results at 7 days postoperatively (16.13 ± 7.10 VS 29.46 ± 9.42; *P* = 0.000*), however, there was no significant difference between the two groups in ODI scores at 6 and 12 months (Table 2).

### JOA scores

There were no significant differences in JOA scores between two groups before the operations (11.8 ± 5.8 VS 12.8 ± 5.8 ; *P*>0.05), At 7 days after the operation, the control group was associated with significantly lower JOA scores (16.3 ± 4.2 vs 18.4 ± 3.4; *P* = 0.041*). The results at 3 months postoperatively were consistent with the results at 7 days postoperatively (21.77 ± 2.66 VS 23.83 ± 1.86 ; *P* = 0.001*), however, there was no significant difference between the two groups in JOA scores at 6 and 12 months (Table 2).

### SF-36 scores

There were no significant differences in SF-36 scores between two groups before the operations. Study group only yielded a significant improvement in SF-36 PF (68.7 ± 14.3vs. 60.0 ± 12.0; *P* = 0.018*), SF-36 GH (68.2 ± 16.5 vs. 58.8 ± 14.8; *P* = 0.030*), SF-36 RE (63.3 ± 33.2 vs 43.6 ± 36.2 ,*P* = 0.038*), SF-36 PCS (62.8 ± 13.9 vs. 54.8 ± 13.3 ; *P* = 0.033*) at 3 months compared to control group. There was no significant difference in other SF-36 items at other follow-up time points between two groups (Table 3).

**Table 3:** Preopreative and follow-up time items of physical scale SF-36 scores

	peropreative	3 months	6 months	1 year
Physical Functioning (P-TLIF)	37.3 ± 28.6	60.0 ± 12.0	70.8 ± 15.3	79.8 ± 11.2
Physical Functioning (Wiltse approach TLIF)	40.7 ± 26.5	68.7 ± 14.3	75.8 ± 13.8	81.5 ± 10.8
*P* Values	0.65	0.018*	0.198	0.567
Role-Physical (P-TLIF)	2.9 ± 8.1	29.8 ± 30.0	62.5 ± 37.6	71.2 ± 28.0
Role-Physical (Wiltse approach TLIF)	5.8 ± 14.2	42.5 ± 30.9	63.3 ± 32.7	69.2 ± 29.1
*P* Values	0.355	0.126	0.930	0.796
Bodily Pain (P-TLIF)	32.7 ± 19.1	70.5 ± 9.3	69.0 ± 10.5	75.4 ± 12.2
Bodily Pain (Wiltse approach TLIF)	37.5 ± 21.9	71.3 ± 11.7	72.2 ± 9.6	72.5 ± 10.8
*P* Values	0.382	0.658	0.238	0.692
General Health (P-TLIF)	45.6 ± 15.2	58.8 ± 14.8	65.4 ± 12.9	73.0 ± 10.1
General Healtl (Wiltse approach TLIF)	48.0 ± 17.6	68.2 ± 16.5	70.1 ± 14.1	72.5 ± 10.8
*P* Values	0.569	0.030*	0.200	0.859
Vitality (P-TLIF)	51.9 ± 16.7	65.6 ± 17.1	75.5 ± 8.9	71.5 ± 12.8
Vitality (Wiltse approach TLIF)	51.8 ± 17.1	69.8 ± 12.2	72.2 ± 8.6	70.3 ± 12.0
*P* Values	0.984	0.284	0.887	0.718
Social Functioning (P-TLIF)	49.6 ± 23.8	63.2 ± 23.6	74.4 ± 21.3	79.1 ± 14.8
Social Functioning (Wiltse approach TLIF)	52.6 ± 21.6	70.7 ± 22.9	78.1 ± 16.4	76.7 ± 14.4
*P* Values	0.621	0.284	0.443	0.544
Role-Emotional (P-TLIF)	38.5 ± 46.8	43.6 ± 36.2	66.7 ± 36.5	93.6 ± 23.1
Role-Emotional (Wiltse approach TLIF)	60.2 ± 14.7	63.3 ± 33.2	70.0 ± 29.5	95.6 ± 19.0
*P* Values	0.151	0.038*	0.707	0.729
Mental Health (P-TLIF)	60.2 ± 14.7	68.2 ± 18.8	70.5 ± 9.9	74.9 ± 6.6
Mental Health (Wiltse approach TLIF)	54.0 ± 20.26	72.9 ± 15.6	72.0 ± 8.8	74.5 ± 8.5
*P* Values	0.205	0.303	0.541	0.850
Physical Component Summary (P-TLIF)	29.6 ± 13.2	54.8 ± 13.3	66.9 ± 16.1	74.8 ± 12.0
Physical Component Summary (Wiltse approach TLIF)	33.0 ± 11.0	62.8 ± 13.9	70.4 ± 14.1	74.2 ± 13.3
*P* Values	0.291	0.033*	0.397	0.862
Mental Component Summary (P-TLIF)	50.0 ± 17.7	60.1 ± 20.2	71.0 ± 13.5	79.8 ± 9.5
Mental Component Summary (Wiltse approach TLIF)	45.2 ± 17.0	69.2 ± 17.0	73.1 ± 10.2	79.3 ± 8.7
*P* Values	0.299	0.073	0.540	0.837

### Evaluation of paravertebral muscle atrophy and fatty infiltration

**Fig. 5:**
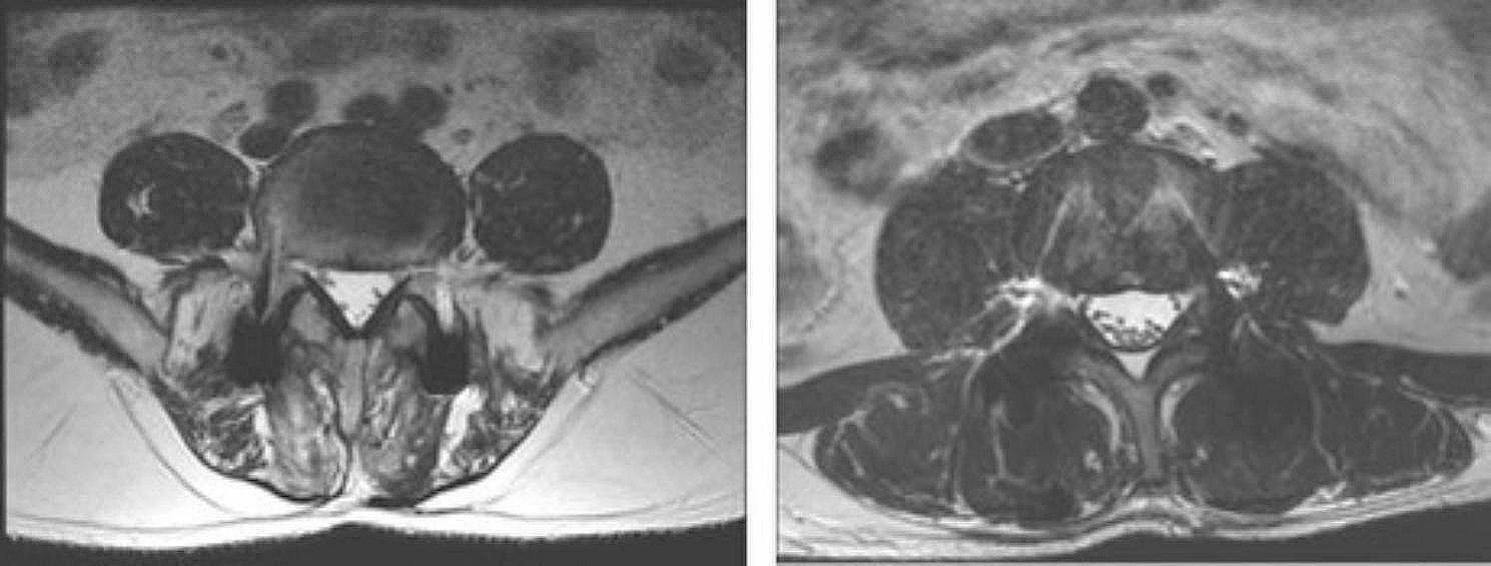
Paraspinal muscles MRI signal 6 months after operation :right is wiltse approach TLIF group left is P-TLIF group

In this study, paraspinal muscle fatty infiltration and fibrosis rates in both groups were comparable before surgery (*P* > 0.05), and only 37 patients (17 in the control group and 20 in the study group) underwent lumbar MRI examination in 6 months after surgery. At six months, paraspinal muscle fatty infiltration and fibrosis in the control group were significantly higher than in the study group ((*P* = 0.008*),(Table 4; Fig. 5).

**Table 4:** MRI signal rank of paraspinal muscles at pre- and 6 months after surgery

	P-TLIF (*n* = 17)				Wiltse approach -TLIF (*n* = 20)				*P* Value
Rank	I	II	III	IV	I	II	III	IV	-
Preoperation	2	9	6	0	2	9	6	3	*P* = 0.399
Postoperation	0	1	9	7	0	9	8	3	*P* = 0.008*

### Surgical complications

There were one case of postoperative cerebrospinal fluid leakage and one case of surgical site infection in the control group, but no severe complication, In the study group, one cases of FBSS and one case of cage displacement were observed. No significant difference in the incidence of complications was observed between the control and study groups (7.7% vs. 6.7%, *P* > 0.05, Table 5).

**Table 5:** Complications at 12-month minimum follow-up

		Wiltse approach TLIF	P-TLIF	
Screw malposition		0	0	
Cage migration		1	0	
CSF leakage		0	1	
Surgical site infection		0	1	
Neurologic defict		0	0	
DEEP venous thrombosis		0	0	
Pulmonary embolus		0	0	
FBSS		1	0	
Total incidence		6.7%	7.7%	
*P* value	*P* > 0.5 *P* > 0.5			

### Intervertebral fusion rate

**Fig. 6:**
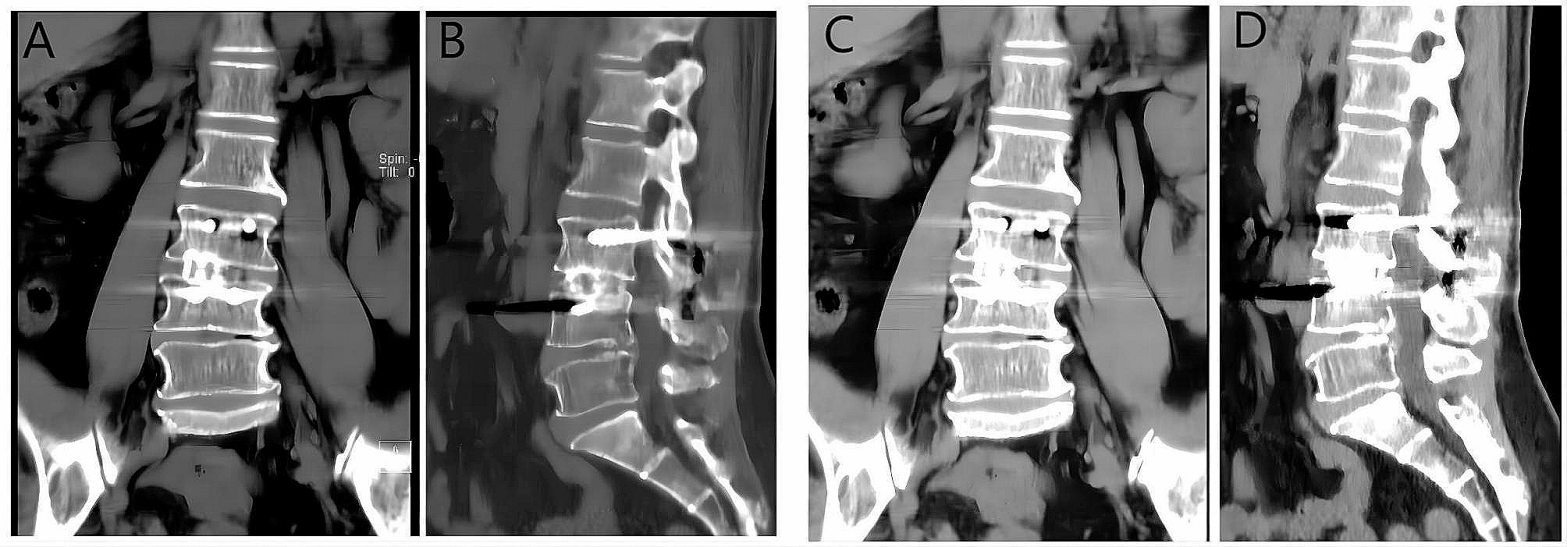
WiltseTLIF and P-TLIF group showed definite bone connection. (**A**) and (**B**): 12 months CT of a Wiltse approach TLIF patient. (**C**) and (**D**): 12 months CT of a P-TLIF patient

There was no significant difference in the intervertebral fusion rate between two groups (*P* = 0.582). The intervertebral fusion rate was 89% in the Wiltse group, and 86% in the P-TLIF group (Table 6; Fig. 6).

**Table 6:** Fusion rate at 12-month minimum follow-up

		Wiltse approach TLIF	P-TLIF	P
Interbody fusion rate, %		89	86	0.582

## Discussion

It is well-established that the P-TLIF technique enables neural canal decompression without exposing the important structures in the spinal canal [12]. However, bilateral paraspinal muscles and require stripping during P-TLIF, which results in paraspinal muscle atrophy, fibrosis, and fat deposition. Emerging evidence suggests that intraoperative paraspinal muscle injury may lead to postoperative pain syndromes [12–15]. In 1968, Wiltse introduced an approach involving leveraging a natural cleavage plane between the multifidus and longissimus muscles to avoid dissecting muscular attachments of the paraspinous musculature to gain entry to the posterior elements [16]. Therefore, using this particular approach leads to improved retention of the multifidus muscle’s physiological functions, maintains stability in the spine, and reduces the incidence of Failed Back Surgery Syndrome (FBSS) [17]. However, there are some limitations in the traditional Wiltse approach TLIF This procedure traditionally uses a laminectomy retractor to expose the pedicle screw entry point, anchored by assistants on the lateral margin of the superior articular process joint while retracting the longissimus muscle laterally. Due to the design flaws in the laminectomy retractor, it is difficult for assistants to use the retractor to pull the multifidus muscle inward and expose the veterbral plate, making it challenging to achieve spinal canal decompression during surgery. Thus, performing spinal canal decompression through traditional Wiltse approaches poses a great challenge for surgeons. To further increase the surgical convenience of this approach, we independently designed a new hand-held retractor system.

### New hand-held retractor assisted Wiltse TLIF can get better perioperative outcomes than P-TLIF

The results of this study showed the novel hand-held retractor assisted Wiltse TLIF is significantly superior to P-TLIF in terms of intraoperative bleeding (79.67 ± 28.59 ml vs. 192.31 ± 59.48 ml, *P* = 0.000*), postoperative drainage (43.33 ± 27.89 ml vs. 285.57 ± 123.05 ml, *P* = 0.000*), mobilization time (4. 1 ± 1.2 d vs. 3.0 ± 0.9 d, *P* = 0.000*), and discharge time (7.7 ± 1.9 d vs. 6.1 ± 1.2 d, *P* = 0.000*), and the results were consistent with previous studies [9, 18]. In the Wiltse TLIF procedure under this novel hand-held retractor system, pedicle screw placement, facet joint resection and interbody bone implantation are conducted under direct vision, which can be easily completed through the natural gap between the longissimus muscles and multifidus muscles, without distraction of the paraspinal muscles, thereby minimizing the extent and duration of muscle distraction compared to the traditional approach, which result in less intraopreative blood lose and pstopreative drainage. In Wiltse TLIF surgery, blood loss is mainly caused by bone surface bleeding after osteotomy and rupture of the spinal venous plexus.

### New hand-held retractor assisted wiltse TLIF more effectively reduce intraoperative paraspianl muscle injury compared to P-TLIF

it is widely acknowledged that CK can be released into peripheral blood during skeletal muscle injury, peaking on day 1 and declining to normal levels on day 5 [19]^20^. Accordingly, peripheral blood CK levels can reflect the degree of intraoperative paraspinal muscle injury. Liu et al. [8, 9, 18] showed that postoperative serum creatine kinase levels were significantly lower in the Wiltse TLIF group compared with the P-TLIF group. In our study, the CK levels in the P-TLIF group at 24 h after surgery (384.10 ± 141.99 U/L) were significantly higher than in the Wiltse TLIF group (532.76 ± 225.76 U/L), indicating that the Wiltse TLIF using hand-held retractors yielded less intraoperative lumbar paraspinal muscle injuryOver the years, MRI has been used to assess the degree of paraspinal muscle degeneration in several studies, the infiltration of fat and connective tissue are mainly manifested as enhanced signals on T2-weighted imaging [20–22]. The traditional posterior approach requires surgeons expose the veterbrae plate and articular process by dissecting the paraspinal muscles on both sides of the spinous process which can result in damage to paraspinal muscles. In our study we found that the paraspinal muscle fatty infiltration and fibrosis in the P-TLIF group were significantly higher than in the Wiltse TLIF using hand-held retractors. This result is consistent with previous study [8]. 

### New hand-held retractor assisted wiltse TLIF has advantage in improving the short-term clinical outcomes

Patient-reported outcome indicators, such as the VAS,ODI and JOA scores, play an irreplaceable role in assessing clinical efficacy [23]. Moreover, previous studies have reported on the correlation between the degeneration of paraspinal muscles after lumbar surgery and these indicators [24]. In our study, the postoperative VAS,ODI and JOA scores in all groups were significantly improved compared with preoperative ones (*P* < 0.05). Similar to Kim’s review [25], postoperative outcomes at or within 6 months were defined as short-term outcomes, and outcomes beyond 1 year were defined as long-term outcomes in this study. Previous studies have substantiated that Wiltse TLIF could improve short-term clinical outcomes [9, 18]. At 7 days after the operation, the Wiltse TLIF group was associated with significantly lower VAS and ODI scores but higher JOA scores, and the results at 3 months postoperatively were consistent with the results at 7 days postoperatively. Although most researchers proved that the Wiltse approach TLIF could improve short-term clinical outcomes, the long-term effects remain subject to debate. It has been reported that patients who underwent Wiltse TLIF have lower ODI and VAS scores one year after surgery compared with P-TLIF [9], and no significant difference in the long-term VAS and ODI scores was present between the two groups [8]. In our study, the VAS,ODI and JOA scores in the two groups were similar one year after surgery.

### Hand-held retractor assisted wiltse TLIF can safely and effectively treat lumbar degenerative disc disease, yielding a similar fusion rate compare to TLIF

The incidence of complications can be used to evaluate the safety profile of a technique [26]. There is a rich literature substantiating that the Wiltse approach TLIF technique is generally limited to a narrow surgical space, which may lead to more complications, such as the dural sac tear and cerebrospinal fluid leakage [26, 27]. Importantly, no dural sac tear was observed in cases that underwent the Wiltse TLIF in our study. In the P-TLIF group, one patient experienced cerebrospinal fluid leakage, while another had a surgical site infection after surgery. In the Wiltse TLIF group, one patient suffered cage displacement 3 months later, but a CT examination one year after surgery showed that cage displacement had fused with the upper and lower endplate. The incidence of complications in the two groups was comparable (control group: 7.7%, study group: 6.7%, respectively). Overall, the results of the current study are consistent with findings reported by Liu et al. [9]. Growing literature suggests that the fusion rate of the intervertebral cage may affect the clinical outcomes of patients [28–31]. The present study found no significant difference in the fusion rate between the two surgical methods at the one-year postoperative, suggesting that both techniques can achieve the expected fusion results (Fig. 6).

### Advantages of this new hand-held retractor system

The hand-held retractor system is specifically designed for Wiltse approach surgery, with a focus on Wiltse TLIF procedures. In the novel hand-held retractor assisted Wiltse TLIF procedure, surgeons can use the articular process retractor to expose the pedicle screw entry point of lumbar veterbrae by clamping its short arm at the basement of the transverse process and the long arm at the lower edge of the transverse process. Additionally, surgeons can also expose the pedicle screw entry point of S1 by clamping its short arm at the lateral margin of the superior articular process and the long arm at the sacrum. After the pedicle screw is inserted, a greater intraoperative field is obtained as surgeons clamp the “U”-shaped head of pedicle screw retractor at the base of the pedicle and pull outward, and the veterbral plate is exposed as assistants use the soft tissue retractor to pull the multifidus inward. The advantages of this retractor system are as follows: (i): the veterbral plate can be exposed by pulling through multifudus muscle inward, making the spinal canal decompression more convenient; (ii): The articular process joint retractor is designed with consideration of the anatomical structure of the surgical field to ensure a stable contact between the retractor and the bone (facet joint, transverse process and sacrum), with minimal risk of injury to these structures. therefore, surgeon can insert the pedicle screw in the meanwhile reduce the incidence of intraoperative accident (such as superior articular process joint fracture and transverse process fracture). (ii). The Wiltse approach typically employs a laminectomy retractor for surgical field exposure. An assistant secures it to retract the longissimus muscle towards the lateral margin.In patients with muscularity or obesity, the multifidus muscle can impede the surgical view. In our study, after the pedicle screw inserted, the surgical field can be exposed by the assistant fixing the pedicle screw retractor on the pedicle screw and pressing the end part of the retractor. It can facilitate the exposure of the vertebral plate, aiding in spinal canal decompression.

### Limitation

The limitations and shortcomings of the present study should be acknowledged. First of all, all cases were performed by one surgeon, which affects the generalizability of our findings. In this study, we did’t set the laminectomy retractor assisted Wiltse TLIF group to further explore the advantage of this novel hand-held retractor system. Further more, There was not only one level fusion surgery patients but also two levels fusion surgery inlcuded which might impact the results of this study. In addition, the sample size was small, which may have affected the reliability of our comparison between Wiltse TLIF and P-TLIF to a certain extent. Moreover, whether the Wiltse approach TLIF can achieve better long-terme effects has not been established. Further studies are warranted to assess whether Wiltse TLIF using hand-held retractors can significantly improve the long-term outcomes compared with P-TLIF.

## Conclusion

Our study showed that the Wiltse approach TLIF can significantly reduce paraspinal muscle injury, postoperative drainage, and intraoperative blood loss, mobilization and discharge time, as well as yield better short-term outcomes compared to P-TLIF.

## Data Availability

The datasets generated during and/or analyzed during the current study are available from the corresponding author on reasonable request.

## Abbreviations

P-/Witlse-/MIS TLIF: Posterior/Witlse/Mini invasive transforaminal lumbar interbody fusion
CT: Computed Tomography
LBP: Low back pain
VAS: visual analog scale
ODI: Oswestry disability index
SF-36: Short-Form 36
CK: Serum creatine kinase
CRP: C-reactive protein
BMI: Body mass index
CSF leakage: Cerebrospinal fluid leakage
FBSS: Failed back surgery syndrome
